# Novel gain of function approaches for vaccine candidate identification in *Burkholderia pseudomallei*

**DOI:** 10.3389/fcimb.2012.00139

**Published:** 2013-01-09

**Authors:** Andrea J. Dowling

**Affiliations:** Biosciences, College of Life and Environmental Sciences, University of ExeterPenryn, UK

**Keywords:** *Burkholderia*, vaccine, melioidosis, novel screening, virulence factors

## Abstract

The Gram-negative bacterium *Burkholderia pseudomallei* is a serious environmental pathogen and the causative agent of the often fatal melioidosis. Disease occurs following exposure to contaminated water or soil, usually through cuts in the skin or via inhalation. However, the underlying mechanisms of pathogenicity remain poorly understood. *B. pseudomallei* is endemic to South East Asia and Northern Australia where infections are associated with antibiotic resistance and high mortality rates. Categorization of the pathogen as a potential biowarfare agent has also made research into vaccine development a high priority. Recent genome-scale screening has produced a large number of putative gene candidates from *B. pseudomallei* with the potential for development into vaccines. This mini-review will discuss the advantages and limitations of this novel approach, how these new techniques can complement existing strategies, and outline aims for future research.

## Introduction

*Burkholderia pseudomallei* is a serious environmental pathogen of man and the causative agent of the often fatal disease melioidosis. Infection occurs following exposure to contaminated water or soil, usually through cuts in the skin or via inhalation, but the underlying mechanisms of pathogenicity of *B. pseudomallei* to humans remain poorly understood (Adler et al., 2009). *B. pseudomallei* is endemic to South East Asia and Northern Australia where infections are associated with both antibiotic resistance and high patient mortality (~50%). The high rates of infection and subsequent mortality make *B. pseudomallei* a high priority for research and vaccine development, as no effective vaccine currently exists. A combination of the lack of vaccine, high infection rate via aerosol inhalation, and limited utility of antibiotics has resulted in classification of this bacterium as a Category B biowarfare pathogen (Rotz et al., 2002).

During the establishment of infection *B. pseudomallei* adheres to, survives and replicates within both host epithelial cells and macrophages. The bacterium is therefore capable of interfering with the host cellular mechanisms that would otherwise destroy it. Known bacterial factors affecting this interaction with host cells include the bacterial capsule, and effectors delivered by the type III and type VI secretion systems (T3SS and T6SS) (Stevens et al., 2003; Shalom et al., 2007; Galyov et al., 2010; Burtnick et al., 2011). Once inside the macrophage the pathogen induces cell fusion leading to the formation of “Multi-Nucleated Giant Cells” or MNGCs, a process key to both intracellular replication and bacterial persistence (Kespichayawattana et al., 2000). Following the invasion of host cells pathogen replication reaches a critical point at which the bacteria induce cell death and are released to establish secondary infections (Adler et al., 2009). Both the intrinsic drug resistance and the intracellular lifestyle of *B. pseudomallei* make successful antibiotic treatment difficult therefore vaccine development is an important strategy against melioidosis.

## Challenging macrophages to identify anti-immune cell factors

**Figure 1:**
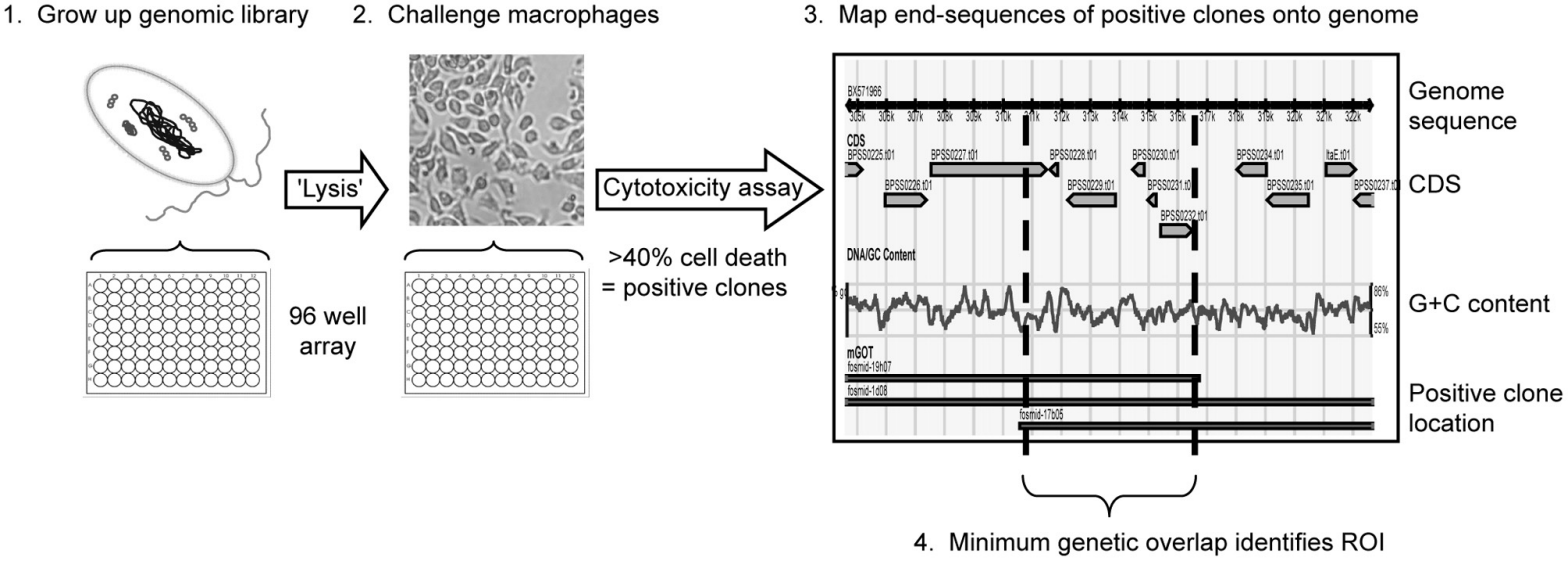
**Brief overview of the anti-macrophage screen workflow.** Recombinant genomic libraries of the sequenced clinical isolate *B. pseudomallei* K96243 were produced in *E. coli* (1), end-sequenced and subsequently screened for cytotoxicity against murine macrophages (2). Library clones shown to reduce macrophage viability by 40% or more are classed as positive and the end sequences of these positive clones are aligned onto the genome sequence (3). Each anti-macrophage locus, or region of interest (ROI), is then described as the minimum region of genetic overlap covered by two or more positive clones (4).

Evidence suggests that macrophages are an essential early defence against *Burkholderia* infection (Breitbach et al., 2006). These innate immune cells are one of the first responding phagocytes to arrive at the site of infection where they phagocytose invading bacteria and (along with other cells) release chemokines and cytokines to govern immune responses and promote adaptive immunity. To establish intracellular infection *Burkholderia* must evade macrophage killing and disrupt the normal cell signaling and regulation processes. In order to detect potential vaccine candidates a simple “gain of function” screen was designed aimed at identifying genes encoding anti-macrophage virulence factors across the *B. pseudomallei* genome (Figure 1). This screen identifies recombinantly expressed genes that equip naïve *Escherichia coli* with anti-macrophage abilities in three different ways: (1) the production of cytotoxic moieties e.g., proteins, secondary metabolites (toxins, effectors), (2) the ability to evade phagocytosis or destruction within the phagolysosome (intracellular survival), and (3) overwhelming e.g., nutrient depletion and starvation (biofilm formation, hydrogen peroxide scavenging) (Dowling et al., 2010).

## Potential vaccine candidates identified

A large proportion of melioidosis vaccines tested to date have focused on the use of live-attenuated strains of *B. pseudomallei*. However, a major issue with live-attenuated vaccines is the risk of reversion to virulence and although they are currently the most effective vaccines studied in mice it is unlikely that they would be used in humans (Sarkar-Tyson and Titball, 2010). Less research has been focused on the development of subunit vaccines (mainly as a putative antigen needs to first be identified) and those investigated are far less effective (Patel et al., 2011). This screen represents a novel method that can be used to directly identify potential recombinant subunit vaccines. The design of the screen means that any active anti-macrophage products identified are successfully expressed within recombinant *E. coli*. Recombinant subunit vaccines are an attractive proposition as they eliminate the safety issues associated with subunit purification from large cultures of the pathogen itself (Liljeqvist and Stahl, 1999).

The original screen revealed over 100 anti-macrophage related loci on both chromosomes 1 and 2 of *B. pseudomallei* strain K96243. Several functional classes were repeatedly identified; putative secondary metabolite synthesis gene clusters (NRPSs and PKSs), signal response and regulation systems (methyl-accepting chemotaxis proteins, two-component sensor systems, AraC and LysR family regulators), phage-related elements, resistance (drugs, metals, reactive oxygen species), adhesion and biofilm formation. The largest number of coding sequences (CDSs) detected however related to toxin/enzyme production (hemolysins, proteases, phospholipases) or transport and secretion (ABC transporters, autotransporters, efflux transporters, and components of Type III and Type VI secretion systems). Examples of putative vaccine candidates from different functional classes discussed in the following text are summarized in Table 1.

**Table 1:** **Examples of putative vaccine candidates identified**.

**Functional class**	**Example vaccine candidate**	**CDS**	**References**
ABC transporter	LPS biosynthesis operon plus *wzt* ABC transporter	BPSL2672-BPSL2688	Garmory and Titball, 2004; Harland et al., 2007b
Autotransporter	BoaA	BPSS0796	Tiyawisutsri et al., 2007; Balder et al., 2010; Lazar Adler et al., 2011
	BoaB	BPSL1705		
Type VI secretion	Hcp proteins (inc. Hcp4, Hcp5, Hcp6) and VgrG proteins	numerous	Pukatzki et al., 2009; Suarez et al., 2010; Burtnick et al., 2011
Toxin	*Burkholderia* Lethal Factor 1	BPSL1549	Cruz-Migoni et al., 2011
Enzyme	Phospholipase D	BPSS1381	McKean et al., 2007; Driskell et al., 2009
	Non-hemolytic phospholipase PLC-3	BPSS0067	Tuanyok et al., 2006
Adhesion	Filamentous hemagglutinin-like	BPSS1721-BPSS1735	Sato et al., 1981; Kimura et al., 1990; Dowling et al., 2010
Secondary metabolism	SylA-like (BylA)	BPSS1263-BPSS1269	Groll et al., 2008; Dowling et al., 2010
Hypothetical protein	Putative toxins	BPSL0590	Dowling et al., 2010
		BPSL0591	

### Transporters and secretion systems

ABC transporter and autotransporter-related proteins are drawing considerable interest as potential vaccine candidates given the surface association of their component proteins which may be exposed to the immune system upon *B. pseudomallei* infection (Garmory and Titball, 2004; Lazar Adler et al., 2011). ABC transporters have a known role in bacterial survival and pathogenicity, 22 of the 105 predicted K96243 ABC transporters were detected by the screen (Harland et al., 2007b; Dowling et al., 2010). Of particular interest among the detected loci are the lipopolysaccharide (LPS) biosynthesis operon and *wzt* ABC transporter (Table 1). This particular finding suggests that an active LPS can be successfully constructed in a recombinant system. Given that LPS elicits strong immune responses this could make it an attractive vaccine candidate. Further, this screen detected 5 out of the 11 documented autotransporters in K96243 and interestingly detects both BPSS0796 (BoaA) and BPSL1705 (BoaB), these trimeric autotransported adhesins are implicated in adhesion to epithelial cells and are shown to have immunogenic properties (Tiyawisutsri et al., 2007; Balder et al., 2010).

Recent advances have been made regarding Type VI secretion machinery proteins; in particular the Hcp family, as subunit vaccine candidates (Burtnick et al., 2011). Three of the six documented *hcp* encoded proteins; Hcp5 (BPSS099), Hcp4 (BPSS0171), and Hcp6 (BPSL3015), were within ROI detected by this screen. However, research so far demonstrates that purified Hcp2 gives the highest-level protection (80%) to BALB/c mice inoculated with a lethal dose of *B. pseudomallei*. Burtnick et al. demonstrated T6SS 1 as a major virulence determinant for *B. pseudomallei* with deletion of *hcp1* resulting in higher LD_50s_ and growth defects compared to wild-type *B. pseudomallei*. Deletion of *vgrG1* from this system also displayed the same results demonstrating its importance in secretion of Hcp1 (Burtnick et al., 2011). VgrG proteins play an important role in host cell cytotoxicity as well as forming the secretion machinery itself and are present on the surface of the pathogen, several VgrG CDSs were identified by the screen (Pukatzki et al., 2009; Suarez et al., 2010).

### Toxins, enzymes, and hypothetical proteins

One of the anti-macrophage regions identified contains a putative Phospholipase D-like protein (BPSS1381) (Dowling et al., 2010). Mutations of Phospholipase D proteins in the pathogens *Corynebacterium pseudotuberculosis* and *Rickettsia prowazekii*, in which the protein is a major virulence factor, have shown potential as live-attenuated vaccines (McKean et al., 2007; Driskell et al., 2009). Further characterization is warranted to determine if this phospholipase D-like protein may be of use as a potential vaccine candidate for *B. pseudomallei*. Another loci of interest encodes the putative toxin PLC-3 (non-hemolytic phospholipase) which has been shown to be strongly upregulated in a hamster model of melioidosis and a mutant displays an LD_50_ significantly higher than wild-type *B. pseudomallei* parent strain (Tuanyok et al., 2006). These data highlight PLC-3 as a potential vaccine candidate warranting further investigation.

Approximately 30% of *B. pseudomallei* genes encode hypothetical proteins whose biological functions are yet to be established. These hypothetical proteins are a potential source of novel virulence factors, and indeed potential vaccine candidates. The anti-macrophage screen promotes the identification of hypothetical proteins with anti-macrophage functions. One positive region described by the screen contained two large adjacent CDSs, BPSL0590 and BPSL0591, encoding hypothetical proteins. The position-specific-iterative BLAST (psi-BLAST) algorithm predicted homology within BPSL0590 and BPSL0591 to known toxins from other pathogens. Both contain domains with homology to the Toxin complexes (Tc's) from *Photorhabdus*, further the predicted N-terminal of BPSL0590 has homology to the *Salmonella enterica* virulence associated protein or SpvB. Macrophages treated with lysate from recombinant *E. coli* library clones expressing BPSL0590 and BPSL0591 show altered actin cytoskeletal morphology, multi-nucleation, and apoptosis illustrating that these may be novel toxins (Dowling et al., 2010).

Recently a novel toxin has been described in *B. pseudomallei* K96243 with similarity to the potent CNF1 toxin from *E. coli* and has been named “*Burkholderia* Lethal Factor 1” or BLF1 (BPSL1549) (Cruz-Migoni et al., 2011). BLF1 has the ability to inhibit helicase activity and thus protein synthesis. BLF1 was previously described as a hypothetical protein given that similarity to CNF1 is based on structural rather than sequence homology. BLF1 mutants were attenuated in their ability to kill mice and purified toxin was lethal upon injection indicating the important nature of this toxin in *B. pseudomallei* virulence (Cruz-Migoni et al., 2011). The BPSL1549 CDS that encodes BLF1 was identified within a positive locus pulled out by the anti-macrophage screen. Of note, the toxin is flanked by two ABC transporter systems (BPSL1545-BPSL1546 and BPSL1548) also contained with the anti-macrophage region of interest that may be involved in secretion of BLF1. The utility of BLF1 as a toxoid vaccine has yet to be explored. There are a further 28 anti-macrophage loci out of the 113 described in the screen which contain at least one hypothetical protein. These are yet to be investigated via detailed protein and structural prediction algorithms to identify active sites or homologies. However, as exemplified by BLF1, such hypothetical proteins warrant further research as a source of potential, currently hidden, virulence factors, and hopefully future vaccine candidates.

### Adhesins

Adhesins make attractive vaccine candidates, as they are surface expressed and likely therefore to be presented to the immune system upon infection. Adhesins are often key virulence determinants allowing the initial attachment of the invading pathogen to host cells. The effectiveness of the Filamentous hemagglutinin (FHA) of *Bordetella pertussis* as an antigen has long been utilized in vaccines against Pertussis (Sato et al., 1981). The region BPSS1721-BPSS1735 identified as an anti-macrophage locus encodes proteins with predicted homology to *B. pertussis* FHA. Macrophages treated with library clones expressing the region showed dramatic actin alterations and apoptotic nuclei. Of note, *B. pertussis* FHA is successful at preventing adherence of the pathogen to the respiratory epithelium when introduced as a protective antigen via intranasal immunization (Kimura et al., 1990). The role of this hemagglutinin-like protein in *B. pseudomallei* infection has not so far been characterized. Determination of whether this FHA homolog may prove useful in protection against melioidosis, in particular disease contracted via inhalation, could help in the formulation of a successful vaccine.

### Secondary metabolites

Many of the anti-macrophage regions identified contained large gene clusters associated with the non-ribosomal peptide synthetase (NRPS) or polyketide synthase (PKS) systems used in the production of peptides and small molecules. The role of such secondary metabolites in virulence is largely undescribed, however, they may be used to promote intracellular survival or production of toxic products and could provide part of the underlying metabolism supporting latent disease. One such anti-macrophage region described by the screen encodes an NRPS gene cluster (BylA) with homology to the SylA (Syringolin A) producing genes from *Pseudomonas syringae* (Dowling et al., 2010). SylA is a proteasome inhibitor and potent cytotoxin (Groll et al., 2008). NRPS and PKS gene clusters and their secondary metabolite products are an unexplored class of vaccine candidates. Purified secondary metabolites may be directly useful as antigens, and there is also the potential for mutating NRPS/PKS gene for use as live-attenuated vaccines.

### Previously evaluated vaccine candidates identified by the screen

The subunit vaccine candidate PotF and the *asd* (aspartate-β-semialdehyde dehydrogenase) gene whose mutation has been investigated for use as a live-attenuated vaccine were re-identified (Harland et al., 2007a; Norris et al., 2011). Asd amino acid products have a role in cell wall biosynthesis and are likely to be necessary for growth and replication (Norris et al., 2011). The *asd* gene may have equipped recombinant library *E. coli* with an increased growth ability enabling them to cause cytotoxicity by overwhelming the macrophages. PotF is an ABC transporter-associated protein involved in putrescine transport documented as necessary for virulence in other bacterial pathogens, putatively involved in host cell attachment and virulence as in a homologous ABC transporter in the plant pathogen *Agrobacterium tumefaciens* and for invasion and intracellular survival in *Salmonella enterica* (Matthysse et al., 1996; Jelsbak et al., 2012). Outer membrane protein A (OmpA) members Omp3 and Omp7 (Hara et al., 2009) and also Omp85 (Su et al., 2010) have been researched as subunit vaccine candidates, however, protection afforded was no greater than with other strategies. Several Omps were identified by this screen, but not those so far tested as candidates. Interestingly, the majority of previously studied vaccine candidates were not re-identified. The main reason for this is that the screen looks specifically for toxicity toward macrophages. Existing subunit candidates were selected for potential immunogenicity and are not necessarily cytotoxic. Most of the genes selected for mutation in the development of live-attenuated vaccines are involved in biosynthetic processes and expression of these in library strain *E. coli* may not result in production of cytotoxic moieties or cytotoxicity as a result of enhanced bacterial growth and overwhelming.

## Advantages and limitations

There are several advantages of using gain of function screens for discovering virulence factors and vaccine candidates. Firstly, they overcome functional redundancy to unmask virulence factors which may be compensated in mutants. *B. pseudomallei* has a large genome and the ability to survive in a wide range of environmental niches. This suggests that functional redundancy is likely among virulence factors with various genes or genetic regions able to compensate mutation to produce successful infection. Secondly, the screens cannot only be used to directly isolate candidates, which may be useful in the development of recombinant subunit vaccines, but also subunit vaccines, DNA vaccines, and live-attenuated vaccines. The screen works by identifying factors that are effective against immune cells via successful recombinant expression in *E. coli*. Production of vaccines via recombinant expression is safer, and more efficient, than purifying from the pathogen itself. Further, the technique narrows down the minimum genetic area needed to produce a response. So far purified proteins have had limited success as vaccines, mainly as this approach requires actual identification of virulence factors or antigenic determinants as candidates first. This problem is tackled by the screening process, which is also able to identify hypothetical proteins with a role in macrophage toxicity. Identifying virulence factors and/or antigens has previously proved difficult for intracellular bacteria (Titball, 2008; Patel et al., 2011). We are currently developing a further novel genomic screen to look for virulence factors involved in intracellular survival and persistence. Thirdly, such screening is rapid and cost-effective, without needing Class III safety facilities and trained staff. Such requirements can restrict the progress of vaccine candidate identification and testing.

One limitation of the screen is the size range of DNA fragments cloned into the BAC and fosmid libraries used (10 kb and 40 kb respectively) therefore any factors above this size will be missed (for example complete type III secretion systems). For successful screening high library quality, in particular good genomic coverage, is necessary to allow repeated identification of cytotoxic clones containing the same genetic region in order to generate confidence in the ROIs identified. The *E. coli* used in screens to date are not modified with for example, regulatory systems, functional secretion systems, or promoters of secondary metabolite production. Although this may limit what is detected, it is also serves as an advantage, as any cytotoxic activity detected is simply a result of successful production by the recombinant library *E. coli* expressing a *B. pseudomallei* DNA fragment. This immediately allows us to narrow down what can be produced in a recombinant system and importantly identify the minimum genetic region necessary to produce bioactivity in such a recombinant system, as would be required for culture and production of a recombinant subunit vaccine. Successful secretion/release from recombinant *E. coli* and effector uptake by macrophages may also limit the factors detected. Some factors may achieve access to macrophages as the cells take in solutes non-specifically from the extracellular media (inoculated with clone lysate preparation) via pinocytosis/macropinocytosis. Further, some viable *E. coli* in the lysate preparation appears necessary for toxicity in some cases.

## Pathways for future research

The number of candidates identified by the gain of function screen is large (>100). Combined approaches correlating datasets obtained from techniques such as *in vivo* expression with this type of recombinant screen will further narrow down key vaccine candidates for follow-up. Use of advanced cytotoxicity screens and high-content phenotype screening will allow detailed functional characterization of candidates. Further investigation of positive regions identified will involve transposon mutagenesis to identify minimum region of activity/genes involved and sub-cloning of these genes to confirm activity. Another useful next step would be to measure the extent of the immune response of macrophages challenged with the anti-macrophage factors identified from the original screen by, for example, assaying nitric oxide production. Screening for adherence and intracellular persistence will identify factors involved in these aspects of *B. pseudomallei* infection. This could prove valuable for development of live-attenuated vaccines for which the issues of safety and potential latency of the mutant *B. pseudomallei* bacteria are of major concern. Introducing additional mutations which will limit or prevent adherence to or persistence within cells in the host could help to make this type of vaccine more viable for use (Patel et al., 2011). These gain of function screens can be used to “mix and match” recombinant vaccine candidates to investigate combinations for possible multivalent subunit vaccines. A combination vaccine composed of polysaccharide and protein presents the most promising formulation for a melioidosis vaccine to date (Patel et al., 2011).

## Concluding remarks

By directly asking macrophages which genomic regions of *B. pseudomallei* express anti-macrophage gene products in a recombinant library system we can begin to isolate virulence factors and potential vaccine candidates. We can explore and characterize the effects of single virulence factors provided that activity can be reproduced in recombinant *E. coli*. This “gain of function” technique can be strengthened by combining with other methods, for example, *in vivo* expression data, providing a powerful tool for discovery, and prioritization of key vaccine candidates for future research.

### Conflict of interest statement

The author declares that the research was conducted in the absence of any commercial or financial relationships that could be construed as a potential conflict of interest.
